# Circulating tumor DNA as a marker of treatment response in BRAF V600E mutated non-melanoma solid tumors

**DOI:** 10.18632/oncotarget.25948

**Published:** 2018-08-24

**Authors:** Lise Barlebo Ahlborn, Ida Viller Tuxen, Florent Mouliere, Savvas Kinalis, Ane Y. Schmidt, Kristoffer Staal Rohrberg, Eric Santoni-Rugiu, Finn Cilius Nielsen, Ulrik Lassen, Christina Westmose Yde, Olga Oestrup, Morten Mau-Sorensen

**Affiliations:** ^1^ The Phase I Unit, Department of Oncology, Rigshospitalet, Copenhagen University, Copenhagen, Denmark; ^2^ Center for Genomic Medicine, Rigshospitalet, Copenhagen University, Copenhagen, Denmark; ^3^ Cancer Research UK Cambridge Institute, University of Cambridge, Cambridge, United Kingdom; ^4^ Department of Pathology, Rigshospitalet, Copenhagen University, Copenhagen, Denmark

**Keywords:** BRAF inhibitor, circulating tumor DNA, mutant allele fraction, early phase study, solid cancer

## Abstract

**Purpose:**

We evaluated longitudinal tracking of BRAF V600E in circulating cell-free DNA (cfDNA) as a marker of treatment response to BRAF inhibitor (BRAFi) combination therapies in non-melanoma solid tumors included in the Copenhagen Prospective Personalized Oncology (CoPPO) program.

**Experimental design:**

Patients with BRAF V600E-mutated tumors were treated with combination therapies including BRAFi. Quantification of mutant cfDNA from plasma was determined and correlated to clinical outcomes. Exome sequencing was performed to identify possible resistance mutations.

**Results:**

Twenty-three patients had BRAF-mutated tumors out of 455 patients included in CoPPO and 17 started BRAFi combination (EGFRi/MEKi) therapy. Tumor responses were achieved in 8 out of 16 evaluable patients and the median overall- and progression-free survival (OS and PFS) was 15 and 4.8 months, respectively. Longitudinal measurements of BRAF V600E-mutant cfDNA indicated disease progression prior to radiological evaluation and a reduction in the mutant fraction of more than 50% after 4 and 12 weeks of therapy was associated with a significantly longer PFS (p=0.003 and p=0.029) and OS (p=0.029 and p=0.017). Furthermore, the baseline mutant fraction and total level of cfDNA positively correlated with tumor burden (p=0.026 and p=0.024). Finally, analysis of cfDNA at progression revealed novel mutations potentially affecting the MAPK pathway.

**Conclusion:**

BRAFi combination therapies showed a response rate of 50% in BRAF V600E-mutated non-melanoma tumors. The fraction of BRAF-mutant cfDNA represent a sensitive indicator for clinical outcomes with plasma collected at week 4 and 12 as crucial time points for monitoring response and disease progression.

## INTRODUCTION

Activating mutations in the *BRAF* gene are present in 5-10% of all human malignancies with the valine-to-glutamic acid substitution at codon 600 (V600E) being the far most common mutation [1]. This mutation appears in a wide range of cancers including melanoma [2], colorectal cancer [3, 4], papillary thyroid cancer [5], non-small cell lung cancer [6, 7], hairy cell leukemia [8], and cholangiocarcinoma [9]. The introduction of specific inhibitors of activated BRAF has greatly improved progression-free survival (PFS) and overall survival (OS) of patients with metastatic melanoma [10]. Dual inhibitor therapies have been successfully tested in malignant melanoma, where combining BRAF inhibitor (BRAFi) treatment with MEK inhibition (MEKi), improved PFS relative to those treated with BRAFi monotherapy [11] and similarly in colorectal cancer adding EGFR inhibitors (EGFRi) to BRAFi [12, 13]. The effect is however temporary as most tumors become resistant to therapy within 6 to 12 months [14, 15]. Consequently, close monitoring of therapy resistance is required but repeated access to tumor tissue is hampered by the invasive nature of tissue biopsies and the associated complications for the patients.

Circulating cell-free DNA (cfDNA) has the potential to monitor therapy response through a simple blood sample, often referred to as a *liquid biopsy*. Tumor specific mutations can be identified in cfDNA and hence presents a minimally-invasive strategy to assess tumor material. In solid tumors, cfDNA has shown great promise in cancer diagnosis, monitoring of therapy, and detection of therapy resistance and clonal evolution (reviewed in [16]). Furthermore, early cancer detection and tumor localization were recently demonstrated based on analyses including cfDNA [17]. In advanced malignant melanoma the *BRAF* V600E mutation can be detected in up to 84% of patients with mutation-positive tumors and the level of mutant DNA (cfBRAFV600E) has been shown to reflect response to targeted treatment including progression and tumor burden [18–20]. Most studies have focused on malignant melanoma and to some extent colorectal cancers [18, 21–25], but little is known about the importance of circulating BRAF DNA in other cancers and how this information is related to BRAFi combination therapy.

In this study, we longitudinally measured the fraction of cfBRAFV600E in plasma samples collected every four weeks from baseline until disease progression from a heterogeneous group of advanced solid cancers included in the Copenhagen Prospective Personalized Oncology (CoPPO) project [26]. The aim was to study the dynamics in circulating BRAF DNA in response to BRAFi combination therapies (MEKi and EGFRi) and correlate these information's to tumor characteristics and clinical outcomes. Furthermore, we performed exome sequencing of cfDNA collected at disease progression across cancer types to identify possible resistance mutations.

## RESULTS

### Patient characteristics

**Figure 1:**
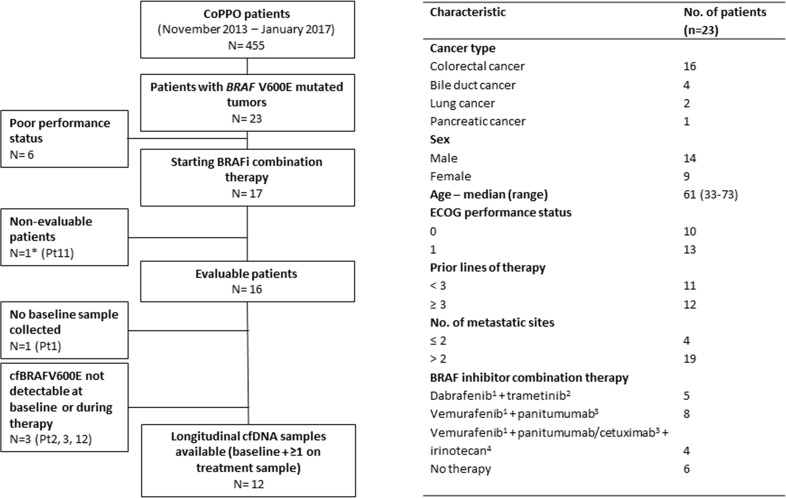
Study outline and baseline characteristics of patients with BRAF V600E mutated non-malignant melanoma tumors Flow diagram (left) showing the number of patients with BRAF V600E- mutated tumors (n=23) and the number of patients with plasma samples collected for cfDNA analysis including baseline analyses (n=16) and longitudinal evaluation (n= 12, requiring a baseline sample and at least one plasma sample after therapy start). One patient was non-evaluable due to premature termination of therapy but the baseline plasma sample from this patient (^*^) was used for statistical analysis on baseline cfDNA levels. Characteristics of the patients with BRAF V600E-mutated tumors are shown in the table (right) including assigned therapy regimens. ^1^BRAF inhibitors administrated were vemurafenib 960 mg twice a day (BID) or dabrafenib 150 BID; ^2^MEK inhibitor was trametinib 2 mg daily (QD); ^3^EGRF inhibitors administrated were cetuximab 500 mg/m^2^ every 14 days or panitumumab 6 mg/kg intravenously (IV) every 14 days; ^4^Irinotecan was administrated at a dose of 180 mg/m^2^ every 14 days.

Mutant BRAF V600E was identified in tumor tissue (fresh biopsy n= 20, FFPE n= 3) in 5% of non-melanoma CoPPO patients (23/455 patients) including colorectal (n=16), bile duct (n=4), lung (Non-Small Cell Lung Cancer, NSCLC) (n=2), and pancreatic cancers (n=1) (Figure 1). Seventeen patients started combination treatment with BRAFi/MEKi (lung and bile duct cancers) or BRAFi/EGFRi with or without irinotecan (colorectal cancer) (Figure 1 and Supplementary Table 2). Six patients never started treatment due to poor performance status and one patient requested early termination of treatment due to toxicity, and was therefore non-evaluable (NE). Sixteen patients were evaluable according to RECIST1.1 and cfDNA was collected at baseline and at least at 4 weeks after starting therapy from 12 of these patients. Four patients had either non-detectable cfBRAFV600E levels using ddPCR (allele fraction, AF < 0.001) or no plasma sample collected at baseline (Figure 1). The latter sample was excluded from the statistical analyses on baseline cfBRAFV600E levels (Figure 3). A total of 124 plasma samples were collected from the 16 evaluable patients with a mean number of 7.75 samples per patient.

**Figure 2:**
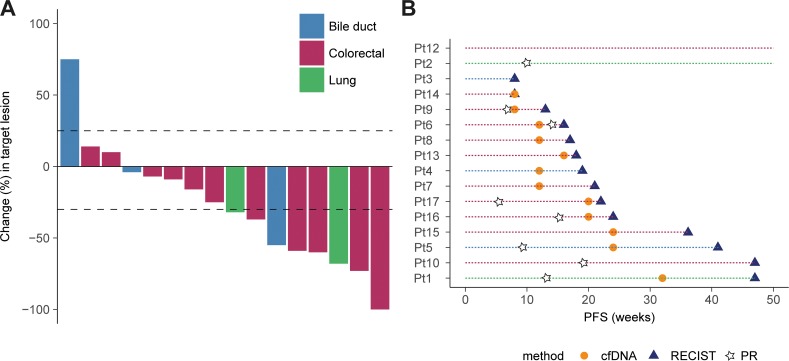
Tumor response and progression assessed by RECIST1.1 and cfDNA **(A)** Waterfall plot showing the maximum %-change in target tumor lesions from baseline to best response according to RECIST1.1. Changes above 20% indicate PD, greater than -30% (line) indicates PR, changes between 20% and -30% indicates SD and tumor reduction of -100% indicate CR. Each patient (n=16) is designated by a colored bar representing the cancer types as shown in the legend. **(B)** Disease progression assessed by cfDNA (orange circles) and RECIST1.1 (blue triangles) for each patient (indicated by a colored horizontal line). The patient ID's are indicated on the y-axis and the x-axis show PFS in weeks. The star symbols indicate the time of best response according to RECIST1.1 for the patients with PR or CR.

**Figure 3:**
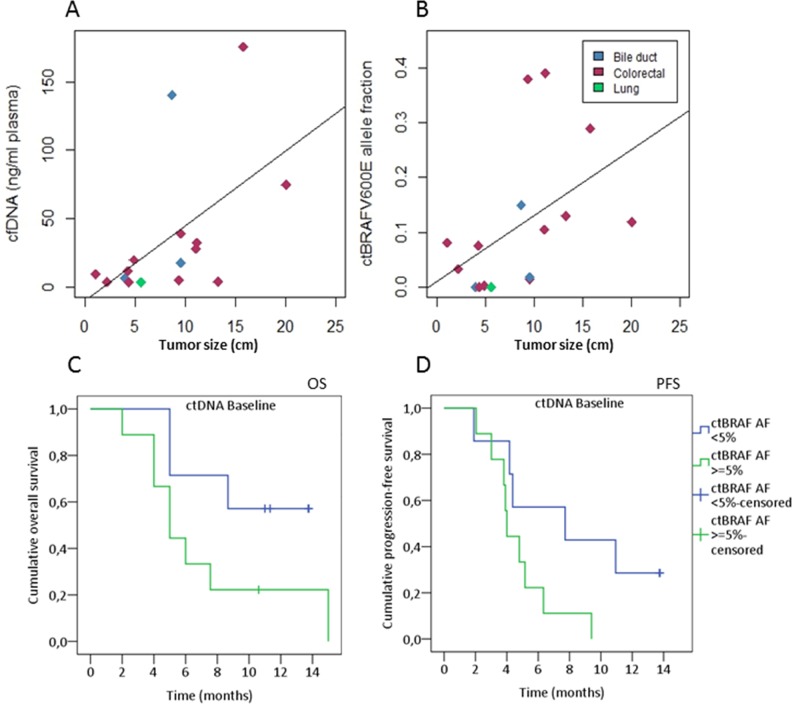
Baseline cfDNA levels correlate with tumor size and survival **(A)** A positive correlation was observed between the sum of target tumor measures according to RECIST 1.1 and total cfDNA levels and cfBRAFV600E allele fraction **(B)**. The Spearman's rank correlation coefficient was 0.55 (p= 0.026) and 0.56 (p= 0.024), respectively. Patients having both a CT scan and a baseline cfDNA sample were included (n=16). Cancer types are indicated by color as shown in the legend. High baseline ctBRAFV600E AF was correlated to shorter OS **(C)** (n=16, p= 0.098, log-rank test) and PFS **(D)** (n=16, *P=* 0.065, log-rank test) in patients with BRAF V600E-mutated cancers stratified on the level of baseline ctBRAFV600E AF threshold of 0.05 (5% allele frequency).

### Tumor response and progression

Thirteen of the 16 evaluable patients (81%) had reductions in their target tumor lesions on CT scans as shown by the waterfall plot (Figure 2A). Eight patients achieved an objective tumor response (CR n=1, PR n=7), seven had SD, and one had PD as best response according to RECIST 1.1. The median PFS and OS were 4.8 and 15 months, respectively (Supplementary Figure 1). Additionally, the PFS ratio was >1.3 in nine patients which means that more than half of the patients experienced >30% longer PFS on BRAFi combinations compared to the most recent treatment (Supplementary Table 2). We also assessed the treatment response in plasma cfDNA and interestingly, changes in BRAF V600E AF compared to baseline, indicated tumor response (decrease in AF) and progression (increase in AF) at 4 and 12 weeks after commencing therapy, respectively (Supplementary Figure 2). In concordance with this observation, patients with a reduction in cfBRAFV600E AF less than 50% after 4 and 12 weeks had a significantly shorter OS and PFS compared to patients with a larger reduction (>50%) from baseline levels (Supplementary Figure 3). Although these analyses were statistically significant, the observations should be assessed in a larger cohort, to confirm the correlations between cfBRAFV600E reduction and survival.

To investigate whether cfDNA was an early marker of tumor progression, we defined progression as the time point when an increase in cfDNA was observed and compared to the date when progression was recorded according to RECIST 1.1. Fourteen patients had tumor progression according to RECIST1.1 within the study period. Increases in cfBRAFV600E AF preceded radiological evidence of progression in 11 out of 16 cases (median 5 weeks, range 2-17 weeks, Figure 2B). The remaining patients were either progression-free at data cut-off (Pt2 and Pt12), had ND levels of cfBRAFV600E at RECIST-defined progression (Pt3 and Pt10) or had unchanged BRAF AF at progression compared to baseline (Pt14). Longitudinal measurements of the mutant AF's are presented for each of the 16 evaluable patients in Supplementary Figure 4.

### Baseline plasma DNA

Baseline plasma samples were available from all BRAF-mutated patients except patient number 1 (Pt1) where no plasma was available before treatment start (Figure 1 and Supplementary Table 2). Plasma cfBRAFV600E was detectable using ddPCR at baseline in 81% (13/16) of the patients. The remaining three patients had undetectable levels of mutant BRAF (AF < 0.001) at baseline and at all measured time points during therapy (Pt 2, 3, 12, Supplementary Figure 4). The total cfDNA level per milliliter plasma (median 14.7 ng/ml, range 3.2 - 175.5, n=16) and cfBRAFV600E AF (median AF 0.079, range ND – 0.39, n=16) indicated a correlation with tumor burden - defined as the sum of tumor measurements according to RECIST 1.1 (Spearman's correlation coefficient, 0.55 and 0.56; *P*<0.05, Figure 3A and 3B). We further tested whether baseline cfBRAFV600E AF ≥0.05 could predict OS and PFS as shown in a similar cohort [27]. Patients with a baseline mutant fraction ≥ 0.05 showed a non-significant trend towards shorter OS and PFS (p=0.098 and p=0.065) compared to those with lower levels (Figure 3C and 3D). Similar results were observed when using the median cfBRAFV600E AF (0.08) as cut-point (data not shown).

### Resistance mechanisms

**Figure 4:**
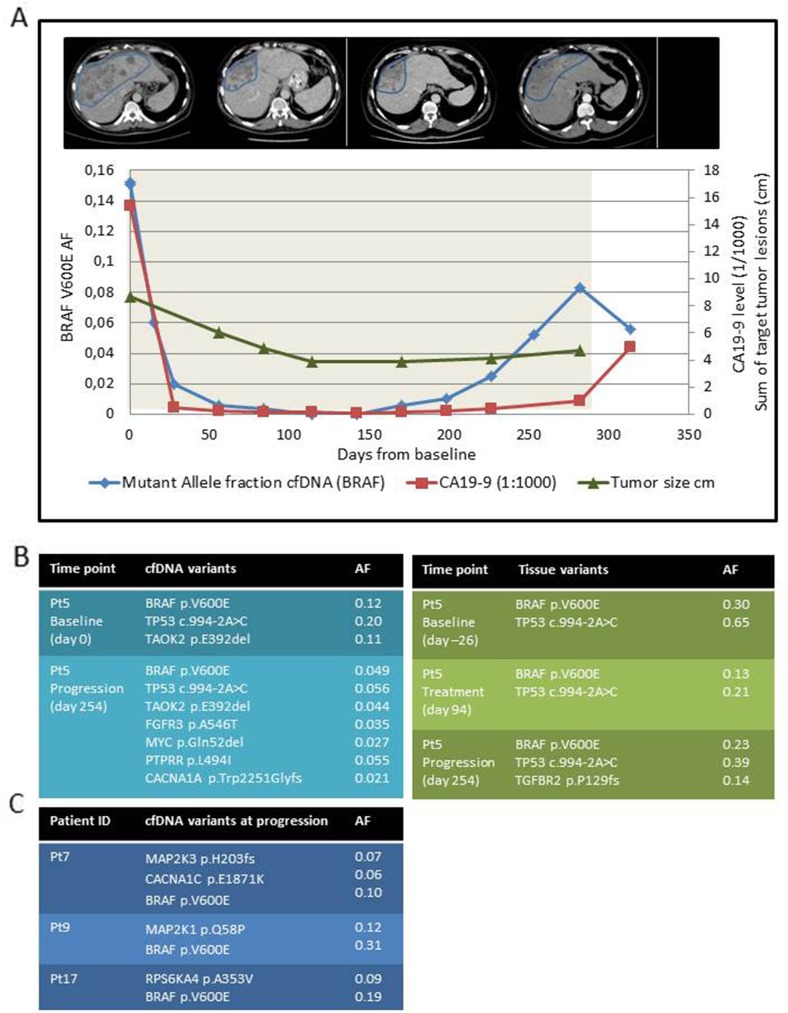
Monitoring tumor response and MAPK-related mutations in response to BRAFi combination therapy **Panel (A)** illustrates the tumor response in a bile duct cancer patient (Pt5). At the top, the tumor response assessed by CT scans marking the area with liver metastasis (blue marking). Below, the changes in the sum of target tumor lesions according to RECIST 1.1(green line), CA19-9 levels (red line) and cfBRAFV600E AF (blue line) are shown. The x-axis indicates the time from therapy start (day 0) until collection of the last plasma sample (day 321) including the day of therapy termination due to progression (day 286, end of grey shading). The primary y-axis indicates the BRAF V600E AF and the secondary y-axis shows both the CA19-9 level (presented as divided by 1000 due to an extreme baseline value of >16000) and the sum of measurable tumor lesions according to RECIST 1.1. **Panel (B)** shows the MAPK-associated variants identified in the three tissue biopsies (green table) and the two plasma samples (blue table). Due to the difference in sequencing depth and therefore mutation fraction cut-offs (tissue AF ≥ 0.10, plasma AF ≥0.02) we were not able to directly compare tissue with plasma mutations. Table **(C)** summarizes the progression-only MAPK-related variants together with the BRAF V600E mutation identified in cfDNA from three colorectal cancer patients with early progression and a high baseline cfBRAFV600E AF.

Characterizing tumor evolution in response to tar-geted therapy is essential for understanding development of resistance. This study included a bile duct cancer patient (Pt5) who achieved a prolonged response, and exome sequencing was performed on three tissue and two plasma samples (Figure 4A–4B). The targeted BRAF mutation and a splice-site-disrupting TP53 mutation were present in all samples analyzed indicating a dominant tumor clone characterized by these two pathogenic mutations. Baseline mutant fractions were higher in the solid and liquid baseline samples compared to the samples collected at progression, likely reflecting the reduced tumor size (Figure 4A). We observed tissue- and plasma-only variants (Figure 4B and Supplementary Table 3) likely illustrating low levels of cfDNA released from a specific tumor clone and the fact that tissue biopsies only represents a fraction of the heterogeneous tumor.

Longitudinal cfBRAFV600E monitoring was compared to images of the liver metastasis, the sum of measurable lesions and cancer antigen 19-9 (CA19-9), and it was evident that, increases in the fraction of cfBRAFV600E indicated tumor growth >100 days (17 weeks) prior to other measures (Figure 4A). Furthermore, whole exome sequencing (WES) and subsequent variant analysis was performedon three cfDNA samples collected at progression from patients with metastatic colorectal cancer (Pt7, 9, and 17). We identified three variants involved in the MAPK pathway (Figure 4C and Supplementary Table 3), which were present at the time of disease progression but not detectable in baseline tumor tissue samples.

## DISCUSSION

Here we show that, the fraction of BRAF V600E in cfDNA can be used to monitor the response to BRAFi combination therapy in non-melanoma cancers and importantly, indicate disease progression median five weeks before radiological evidence. Longitudinal BRAF monitoring showed that, a reduction in AF at 4 and 12 weeks after commencing treatment was significantly associated with longer PFS and OS, indicating that an early drop in cfBRAFV600E levels could be predictive of survival when monitoring advanced stage cancers using cfDNA. Of note, two patients with prolonged survival (Pt2 and Pt12) had non-detectable (ND) cfBRAFV600E levels at baseline and throughout therapy indicating, that ND levels could be a possible prognostic marker as previously suggested [18, 24]. However, these observations are promising, larger cohort studies are needed to further validate the findings. Tumor responses were also evaluated according to RECIST1.1 and a response rate of 50% was achieved across tumor types with median PFS and OS of 4.5 and 15 months, respectively, which are comparable with previous studies [13, 28, 29]. Interestingly, we observed a partial tumor response in both patients with NSCLC and a prolonged PFS and OS compared to previously reported (median PFS 9.7 months) [30].

Despite the small cohort, baseline measurements showed a positive correlation between tumor burden and the levels of total cfDNA and cfBRAFV600E AF. Furthermore, a high baseline BRAF-fraction indicated shorter OS and PFS, an observation that was in agreement with previous reports [21, 24, 25, 27, 31–34]. In addition, we observed a high concordance (83%) in BRAF-mutation status between tumor tissue and cfDNA possibly reflecting the advanced disease status and the use of fresh biopsies instead of archival tumor material [35, 36]. Previous reports have shown a concordance ranging from 57-84% primarily from melanoma studies [12, 18, 20, 21, 24, 32, 37, 38].

This cohort was ideal for evaluating cfBRAFV600E as a marker of therapy response and resistance in non-melanoma cancers, as the cohort included extensive plasma samples and most patients developed disease recurrence within a short timescale. However, our study had some limitations including a small cohort size, heterogeneity of cancer types and treatments. Furthermore, the non-invasive follow-up strategy included only the BRAF V600E mutation which is clinically relevant to only a subset of patients and complementary NGS analysis covering other cancer hotspot genes should be considered for early discovery of driver and resistance alterations.

Resistance to BRAFi therapy constitutes a major challenge and mutations leading to re-activation of the MAPK-pathway have been described in both malignant melanoma and colorectal cancers. Investigating a broad list of 278 MAPK-related genes in cfDNA collected at progression, identified 11 variants (including BRAF V600E), of which six where novel variants and five were previously described in e.g. the COSMIC database. Functional studies are needed to clarify the molecular effect of these variants. Patient Pt9 had a high frequency *MEK1* variant (c.173A>C, p.Q58P, Table S3) located in a conserved region where several alterations have been reported in BRAFi-resistant melanoma samples [39] indicating that, this region might be involved in resistance but functional tests are required. Additionally, WES of multiple tissue and plasma biopsies from a bile duct cancer patient (Pt5) illustrated a dominant tumor clone characterized by a pathogenic TP53 mutation and the treatment target BRAF V600E detected in all solid and liquid biopsies, possibly explaining the good response observed for this patient. Interestingly, progression-only variants were identified in both tissue and plasma DNA but none of the variants were overlapping, possibly illustrating the challenges associated with tumor clonality or differences in sequencing depth and thus allele frequency.

In conclusion, our study indicates that BRAFi combination therapy is effective in advanced, solid non-melanoma BRAFV600E-mutated cancers and cfBRAFV600E may be used as an early indicator of response and progression.

## MATERIALS AND METHODS

### Patients and sample collection

Fresh tumor biopsies were collected and molecularly characterized in the CoPPO study (NCT02290522) as previously described [26]. Formalin-fixed paraffin-embedded (FFPE) archival tumor tissue or plasma cfDNA was examined in cases where tumors were non-accessible for biopsy. All patients had metastatic solid tumors and exhausted treatment options. Studies were conducted in accordance with the Declaration of Helsinki and written informed consent was obtained for all patients (Danish Ethical Committee, file number: 1300530). Patients were allocated to targeted treatment according to recommendations made by a multidisciplinary tumor board. Computed Tomography (CT) scans were performed every eight weeks and tumor responses were evaluated according to Response Evaluation Criteria In Solid Tumors (RECIST) 1.1 [40] and classified as complete response (CR), partial response (PR), stable disease (SD) or progressive disease (PD). The PFS ratio was calculated as PFS on BRAFi combination treatment divided by PFS on most recent treatment. A PFS ratio >1.3 was reported because an improvement in PFS > 30% has been described to be a clinical meaningful cut-off [41]. Plasma samples for cfDNA analysis were collected immediately before treatment initiation (within 24h), longitudinally every four weeks, and if possible, at progression or after treatment termination.

### DNA extraction and quantification

Peripheral blood was collected in BCT tubes (Streck Laboratories, Omaha, NE, USA) as previously described [42]. Circulating DNA was extracted from 2-4 ml plasma using the QIAsymphony Circulating DNA Kit (Qiagen, Hilden, Germany) according to the manufacturer's instructions using an elution volume of 60 μl. Extracted cfDNA was stored at -20°C until further use. DNA quantification was performed using a dsDNA HS Assay Kit (Thermo Fisher Scientific, Waltham, MA) on a Qubit Fluorometer (Thermo Fisher Scientific, Waltham, MA) and the concentration of cfDNA per milliliter of plasma was calculated for each time point.

### Droplet digital PCR

Mutant BRAF V600E was quantified using the QX200 droplet digital PCR (ddPCR) system from Bio-Rad (Bio-Rad/Molecular MD, California, USA). Dual labeled (FAM or HEX) fluorescent probes for BRAF V600E and the wild type loci were used (Bio-Rad, V600E; cat no. 10031246 dHsaCP2000027, WT for V600E; cat no. 10031249 dHsaCP2000028). PCR reaction mixtures were run and subsequently analyzed using the QX200 reader, according to manufactures instructions. The mutant allele fraction (AF, mutant counts/total DNA counts (mutant + wild type)) was estimated using QuantaSoft v.1.7.4 software from Bio-Rad. Mutant AFs ≥0.001 (0.1%) were detected requiring three or more mutation-positive droplets per well as recommended by the company. An increase in cfBRAFV600E AF was recorded if the AF increased from non-detectable to detectable levels (AF ≥ 0.001) or increased in two consecutive samples unless it was the last plasma sample collected prior to termination of treatment due to clinical progression.

### Exome sequencing

Whole exome sequencing was performed on plasma cfDNA samples (Pt5, 7, 9 and 17) obtained at disease progression from patients with a high AF of cfBRAFV600E. Patient tumor samples were also analyzed by WES as part of the CoPPO study [26]. DNA libraries were constructed from 10 ng cfDNA using NEBNext Ultra II DNA Library Prep kit for Illumina protocols (version July 2016, New England Biolabs). Hybridization-based exome capture was performed according to the manufacturer's instructions using the SeqCap EZ MEDExome (Roche NimbleGen Inc.). The samples were pooled and sequenced on the Illumina NextSeq platform to a minimum average coverage >50 x. Analysis of WES data from both tumor tissue DNA and cfDNA was performed using the same pipeline including the Qiagen software Biomedical Genomics Workbench version 3.0 and Ingenuity Variant Analysis version 5.1. Briefly, confident calls originating from bidirectional sequence reads were filtered by a phred quality score ≥30, read depth of ≥20, and allele frequency ≥5%. Tumor-specific variants were selected by subtracting the germline variants which were available through the CoPPO project [26]. Ingenuity Variant Analysis was used to identify coding or splice site disrupting variants (+/- 2 base pairs from exon-intron boundaries). Common variants reported with an allele frequency >1% in the 1000 genomes project, the NHLBI ESP exomes, or the ExAC frequency were removed, unless the variant was established as a common pathogenic variant according to the Ingenuity Knowledge database. Variant analysis was broadly focused on the MAPK pathways using a comprehensive gene list from Gene Set Enrichment Analysis (GESA) including genes from the classical MAP kinase pathway as well as the JNK/p38 and ERK5 pathways (M10792; KEGG_MAPK_SIGNALING_PATHWAY) [43, 44](Supplementary Table 1). In addition, an extended list of cancer-associated variants identified by exome sequencing is included in (Supplementary Table 4). The variant lists were generated using the Ingenuity Variant Analysis software.

### Statistical analyses

Overall survival (OS) and PFS was estimated using Kaplan-Meier statistics [45] and a log-rank test was used to compare OS and PFS among patient groups. PFS was defined, as the time from treatment start to disease progression (according to RECIST 1.1 or clinical evaluation) or death. Overall survival was defined as the time from treatment start to date of death. Patients that were progression-free or still alive at the day of data cut-off (24 October 2017) were censored in the survival analyses. The Spearman rank correlation analysis was used to compare tumor and plasma measures. All analyses were two-sided, and *P*-values <0.05 were considered statistically significant. Statistical analyses were performed using IBM Statistics SPSS (version 22) and R (version 0.99.903).

## SUPPLEMENTARY MATERIALS FIGURES AND TABLES







## Abbreviations

cfDNA: Cell-free DNA
ddPCR: Droplet digital PCR
NGS: Next generation sequencing
BRAFi: BRAF inhibitor
MEKi: MEK inhibitor
EGFRi: EGFR inhibitor
RECIST: Response evaluation criteria in solid tumors
CR: Complete response
PR: Partial response
SD: Stable disease
PD: Progressive disease
BID: twice a day
QD: Daily
AF: Allele fraction.
