# Supplementary material for: Circulating tumor DNA as a marker of treatment response in BRAF V600E mutated non-melanoma solid tumors
Source: Oncotarget. 2018 Aug 24;9(66):32570–9. doi: 10.18632/oncotarget.25948 (PMC6135692; doi:10.18632/oncotarget.25948)
Supplement: Supplementary file 1 [file oncotarget-09-32570-s001.pdf]

Circulating tumor DNA as a marker of treatment response in BRAF V600E mutated non-melanoma solid tumors

SUPPLEMENTARY MATERIALS

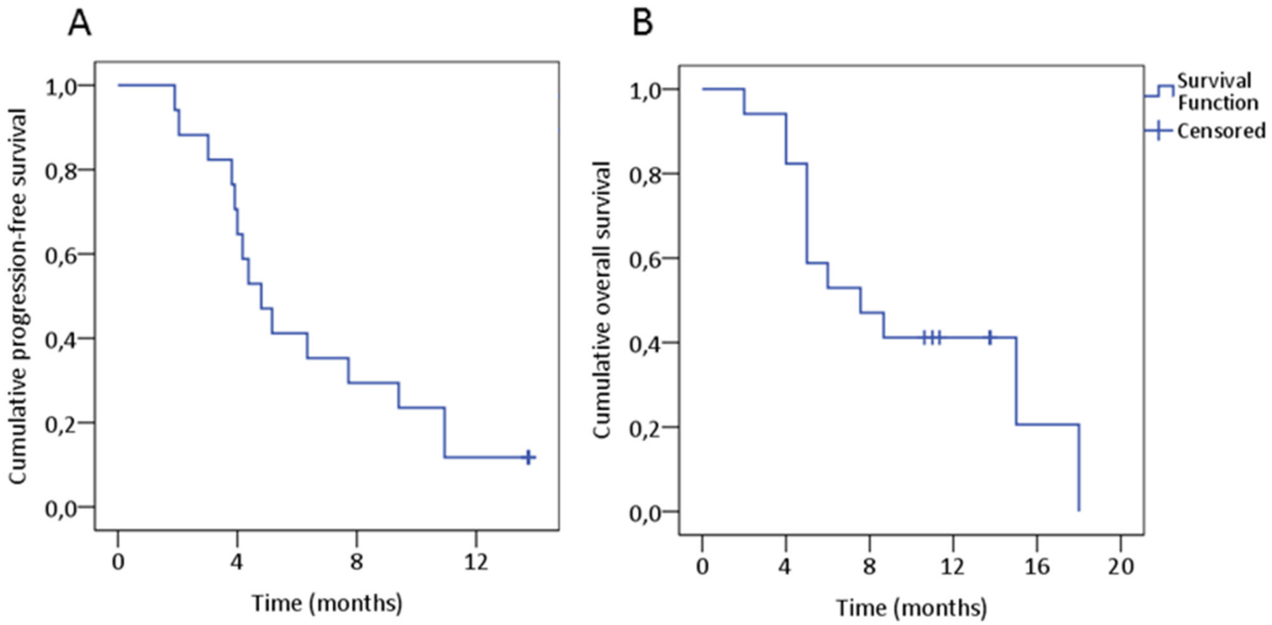

Supplementary Figure 1: Survival in the non-melanoma cohort treated with BRAFi combination therapy. (A) Cumulative PFS, median PFS 4.8 months (Kaplan-Meier statistics, 95% confidence interval (CI): 3.2 – 6.3 months, stand. error: 0.79, n= 17 (5 censored)). **(B)** Cumulative OS, median OS was 15 months (95% CI: 3.3 – 26.7 months, stand. error: 5.9, n= 17 (7 censored)).

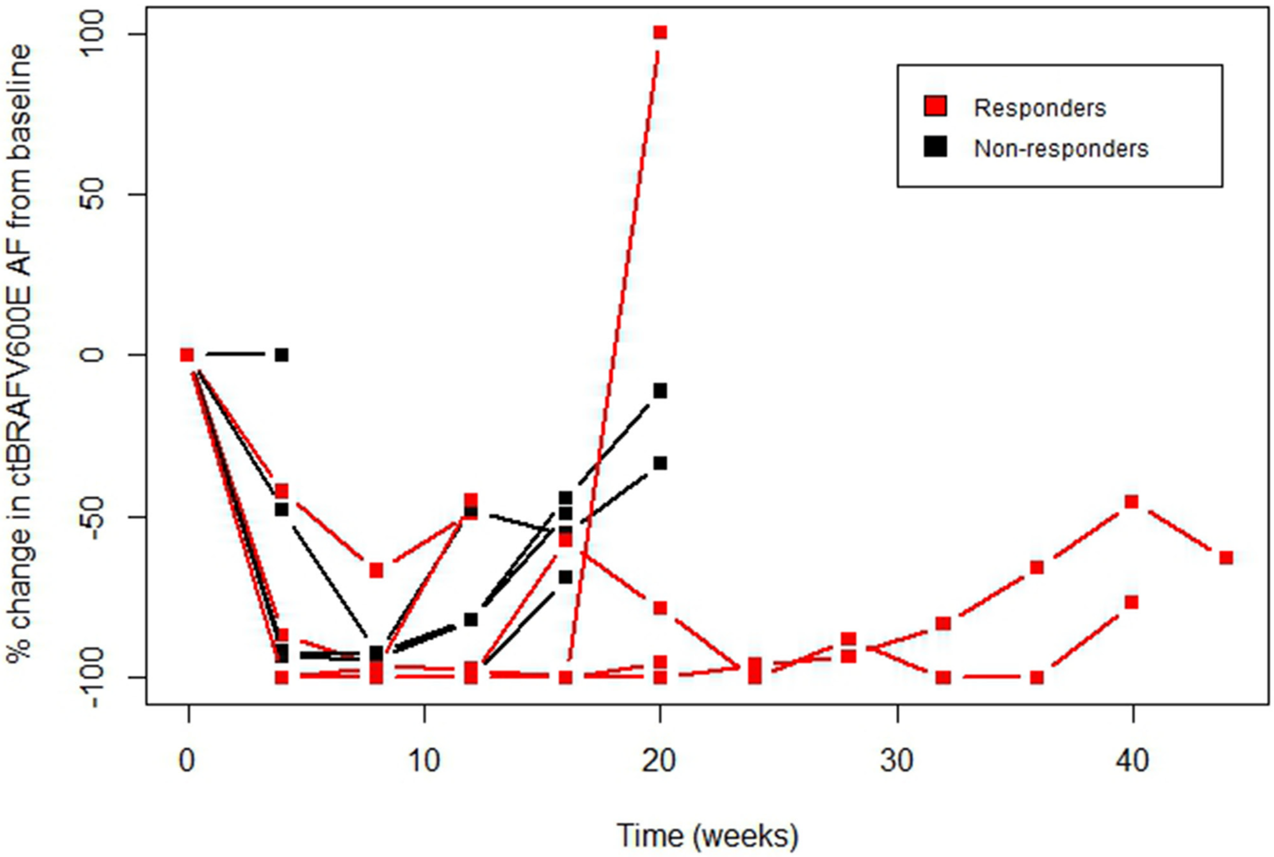

Supplementary Figure 2: Change in ctBRAFV600E AF from baseline in response to BRAFi combination therapy. Serial plasma cfDNA samples were analyzed for the fraction of BRAF V600E using the Bio-Rad QX200 ddPCR system (AF ≥ 0.001) and provided software (QuantaSoft v.1.7.4), and plotted as the percentage change in AF from baseline levels. Longitudinal measures are plotted for responders (PR or CR according to RECIST 1.1, n=6, red lines) and non-responders (PD or SD, n= 6, black lines).

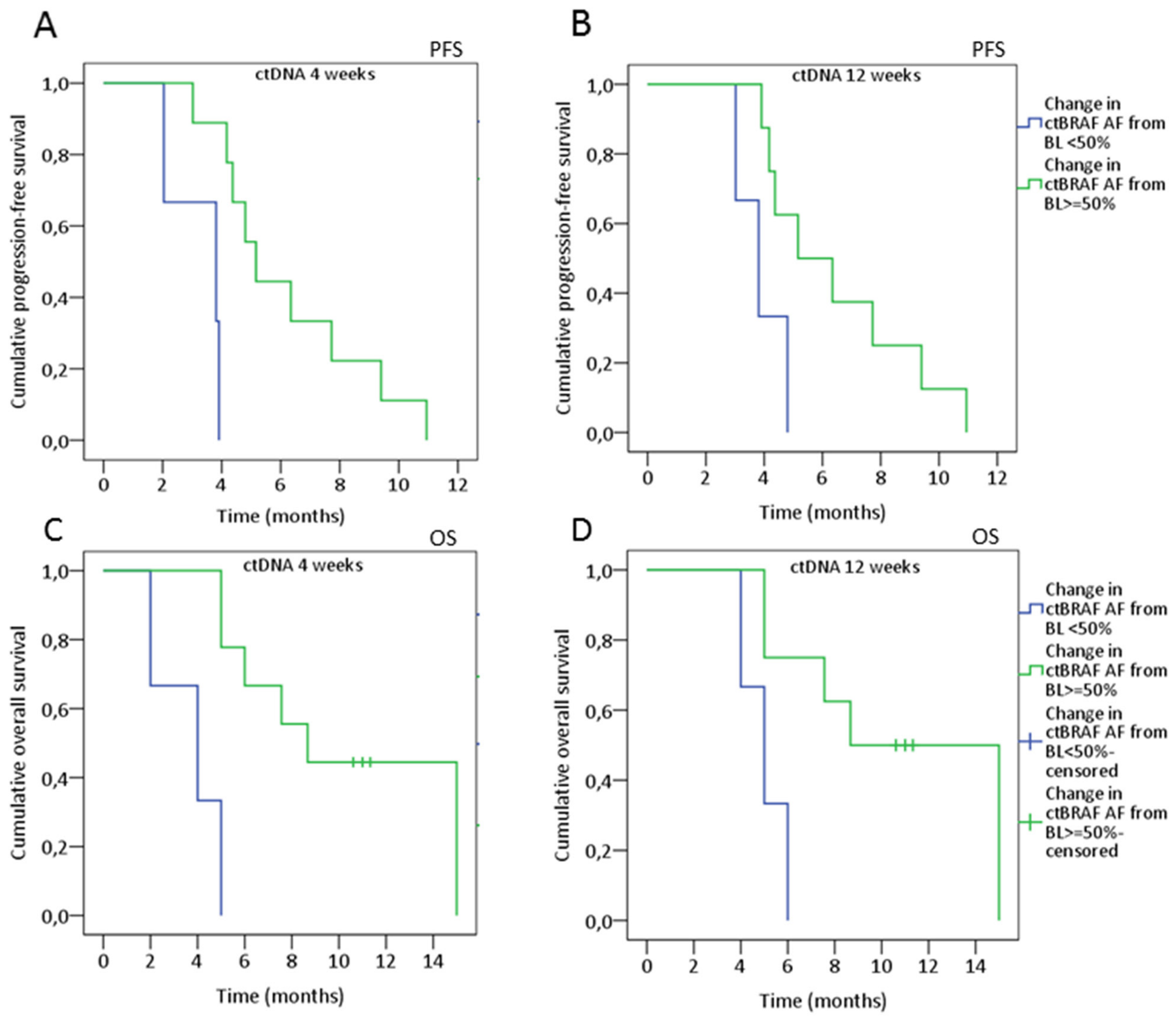

Supplementary Figure 3: The median PFS **(A, B)** (P= 0.003, P= 0.029, log-rank test) and OS **(C, D)** (P= 0.002 and P= 0.017, log-rank test) of patients (n=12) with a change in ctBRAFV600E AF from baseline of >= 50% at 4 and 12 weeks of therapy, respectively, was longer than that of patients with a smaller change. Survival analyses were performed using Kaplan-Meier statistics and included patients with longitudinal cfDNA samples available as shown in Figure 1.

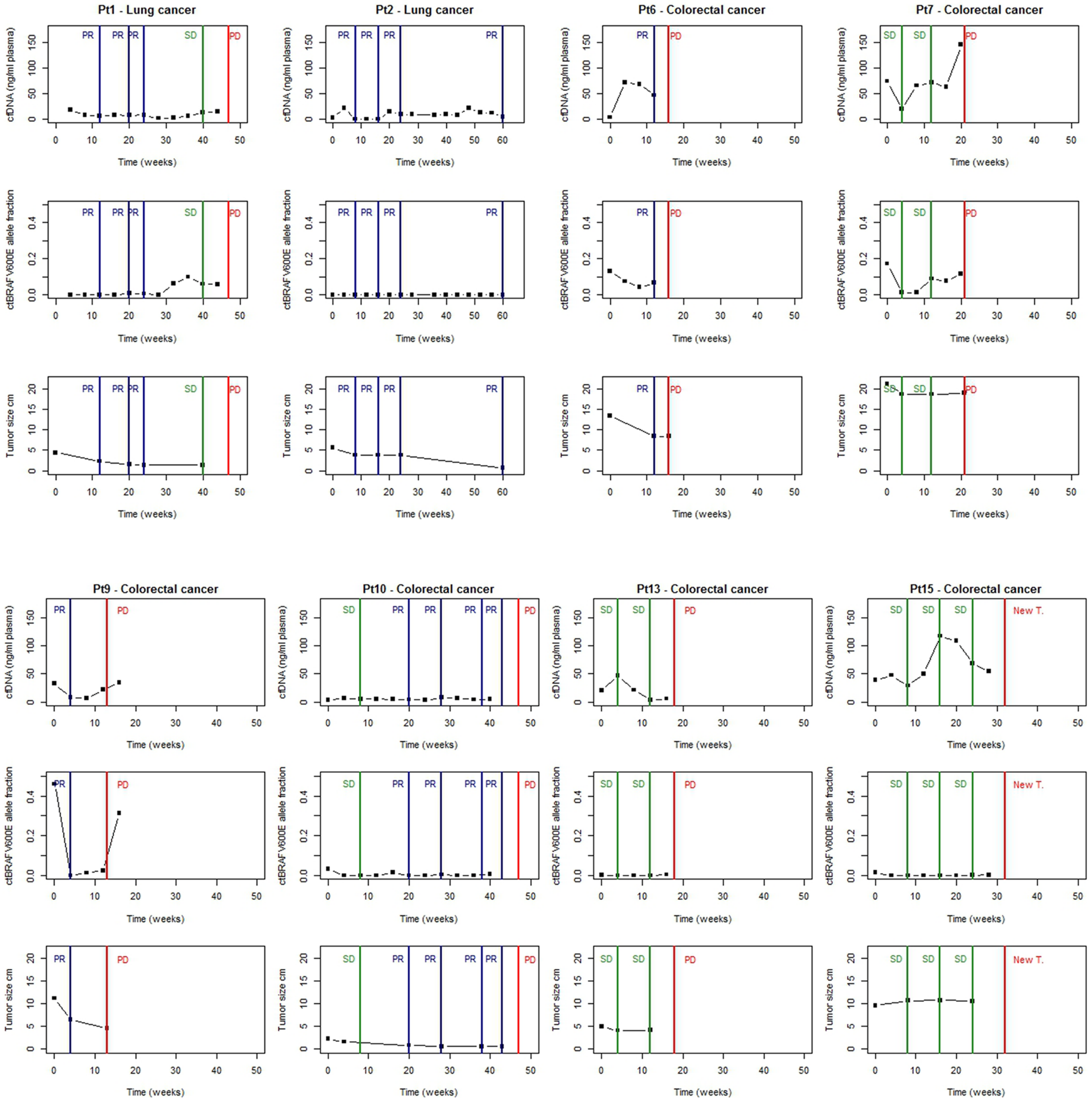

Supplementary Figure 4: Time courses of total cfDNA level (ng/ml plasma), BRAF V600E AF in ctDNA, and sum of target tumor lesions according to RECIST 1.1 (y-axis) in patients with detectable ctBRAFV600E (n= 13) or non-detecable levels at baseline and throughout therapy (n= 3). Time in weeks is shown on the x-axis.

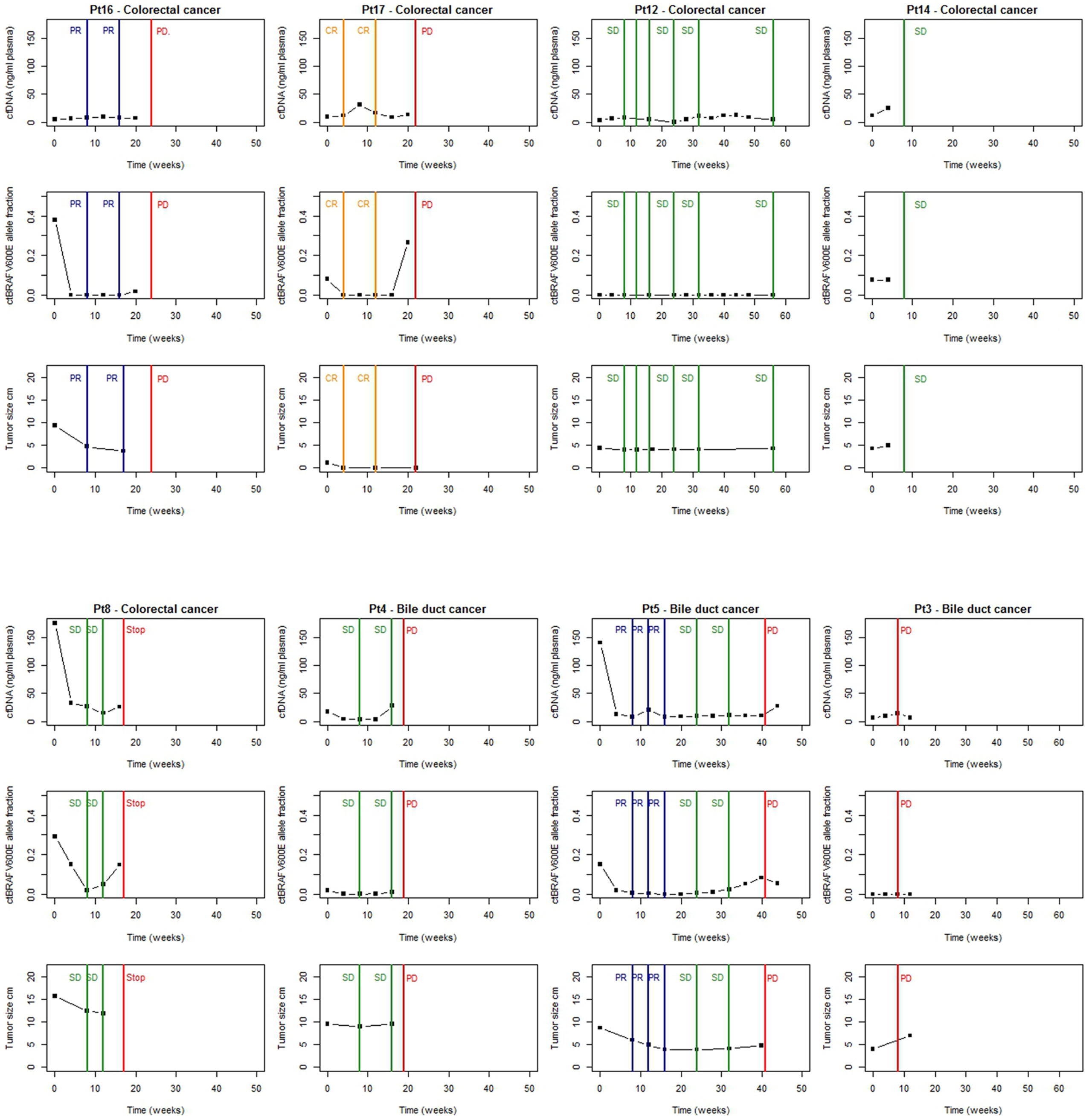

Supplementary Figure 4: (Continued) Time courses of total cfDNA level (ng/ml plasma), BRAF V600E AF in ctDNA, and sum of target tumor lesions according to RECIST 1.1 (y-axis) in patients with detectable ctBRAFV600E (n= 13) or non-detecable levels at baseline and throughout therapy (n= 3). The mutant AF is defined as the fraction of mutated alleles divided by the total number of alleles calculated by the QuantaSoft software. RECIST 1.1 responses are maked with colored lines: CR: Orange; PR: Blue; SD: Green; PD: Red.

Supplementary Table 1: MAPK-signaling-related genes available through Gene Set Enrichment Analysis (GESA) (search term: KEGG_MAPK_SIGNALING_PATHWAY, n=278)

AKT1	CACNB2	DUSP14	FGF17	GADD45B	MAP2K1	MAPK10	NF1	PLA2G2C	PRKX	RPS6KA4	TRAF2
AKT2	CACNB3	DUSP16	FGF18	GADD45G	MAP2K2	MAPK11	NFATC2	PLA2G2D	PTPN5	RPS6KA5	TRAF6
AKT3	CACNB4	DUSP2	FGF19	GNA12	MAP2K3	MAPK12	NFATC4	PLA2G2E	PTPN7	RPS6KA6	ZAK
ARRB1	CACNG1	DUSP3	FGF2	GNG12	MAP2K4	MAPK13	NFKB1	PLA2G2F	PTPRR	RRAS	
ARRB2	CACNG2	DUSP4	FGF20	GRB2	MAP2K5	MAPK14	NFKB2	PLA2G3	RAC1	RRAS2	
ATF2	CACNG3	DUSP5	FGF21	HRAS	MAP2K6	MAPK3	NGF	PLA2G4A	RAC2	SOS1	
ATF4	CACNG4	DUSP6	FGF22	HSPA1A	MAP2K7	MAPK7	NLK	PLA2G4B	RAC3	SOS2	
BDNF	CACNG5	DUSP7	FGF23	HSPA1B	MAP3K1	MAPK8	NR4A1	PLA2G4E	RAF1	SRF	
BRAF	CACNG6	DUSP8	FGF3	HSPA1L	MAP3K11	MAPK8IP1	NRAS	PLA2G5	RAP1A	STK3	
CACNA1A	CACNG7	DUSP9	FGF4	HSPA2	MAP3K12	MAPK8IP2	NTF3	PLA2G6	RAP1B	STK4	
CACNA1B	CACNG8	ECSIT	FGF5	HSPA6	MAP3K13	MAPK8IP3	NTF4	PPM1A	RAPGEF2	STMN1	
CACNA1C	CASP3	EGF	FGF6	HSPA8	MAP3K14	MAPK9	NTRK1	PPM1B	RASA1	TAB1	
CACNA1D	CD14	EGFR	FGF7	HSPB1	MAP3K2	MAPKAPK2	NTRK2	PPP3CA	RASA2	TAB2	
CACNA1E	CDC25B	ELK1	FGF8	IKBKB	MAP3K3	MAPKAPK3	PAK1	PPP3CB	RASGRF1	TAOK1	
CACNA1F	CDC42	ELK4	FGF9	IKBKG	MAP3K4	MAPKAPK5	PAK2	PPP3CC	RASGRF2	TAOK2	
CACNA1G	CHP	FAS	FGFR1	IL1A	MAP3K5	MAPT	PDGFA	PPP3R1	RASGRP1	TAOK3	
CACNA1H	CHP2	FASLG	FGFR2	IL1B	MAP3K6	MAX	PDGFB	PPP3R2	RASGRP2	TGFB1	
CACNA1I	CHUK	FGF1	FGFR3	IL1R1	MAP3K7	MECOM	PDGFRA	PPP5C	RASGRP3	TGFB2	
CACNA1S	CRK	FGF10	FGFR4	IL1R2	MAP3K8	MEF2C	PDGFRB	PRKACA	RASGRP4	TGFB3	
CACNA2D1	CRKL	FGF11	FLNA	JMJD7	MAP4K1	MKNK1	PLA2G10	PRKACB	RELA	TGFBR1	
CACNA2D2	DAXX	FGF12	FLNB	JUN	MAP4K2	MKNK2	PLA2G12A	PRKACG	RELB	TGFBR2	
CACNA2D3	DDIT3	FGF13	FLNC	JUND	MAP4K3	MOS	PLA2G12B	PRKCA	RPS6KA1	TNF	
CACNA2D4	DUSP1	FGF14	FOS	KRAS	MAP4K4	MRAS	PLA2G1B	PRKCB	RPS6KA2	TNFRSF1A	
CACNB1	DUSP10	FGF16	GADD45A	LAMTOR3	MAPK1	MYC	PLA2G2A	PRKCG	RPS6KA3	TP53	

Supplementary Table 2: Survival and baseline tumor and cfDNA measures for patients starting BRAFi combination therapy

Patient ID	Cancer subtype	Therapy	PFS (months)	OS (months)	Best response	Sum of target lesions (cm)	cfDNA BL (ng/ml)	ctBRAFV600E BL freq. (%)	PFS ratio
**Pt1**	Lung cancer	BRAFi + MEKi	10.9	18.0	PR	4.4	No plasma	No plasma	4.6
**Pt2**	Lung cancer	BRAFi + MEKi	13.8*[F]	13.8*[F]	PR	5.6	3.2	ND	7.6*[F]
**Pt3**	Bile duct cancer	BRAFi + MEKi	1.9	5.0	PD	4	6.3	ND	0.3
**Pt4**	Bile duct cancer	BRAFi + MEKi	4.4	5.0	SD	9.6	17.7	1.8	0.4
**Pt5**	Bile duct cancer	BRAFi + MEKi	9.4	15.0	PR	8.7	140.3	15.2	3.4
**Pt6**	Colorectal cancer	BRAFi + EGFRi + Iri	3.8	4.0	PR	13.3	3.75	13.0	1.8
**Pt7**	Colorectal cancer	BRAFi + EGFRi	4.8	5.0	SD	20.1	74.6	11.9	1.1
**Pt8**	Colorectal cancer	BRAFi + EGFRi	3.9	5.0	SD	15.8	175.5	29.1	2.2
**Pt9**	Colorectal cancer	BRAFi + EGFRi	3.0	6.0	PR	11.2	32.4	38.8	0.9
**Pt10**	Colorectal cancer	BRAFi + EGFRi	10.9	11.0	PR	2.2	3.6	3.3	1.6
**Pt11**	Colorectal cancer	BRAFi + EGFRi	4.0 (NE)	4.0	NE	11.1	27.9	10.5	1.5
**Pt12**	Colorectal cancer	BRAFi + EGFRi	13.7*[F]	13.7*[F]	SD	4.4	3.3	ND	0.9*[F]
**Pt13**	Colorectal cancer	BRAFi + EGFRi	4.2	8.7	SD	4.9	19.8	0.2	2.0
**Pt14**	Colorectal cancer	BRAFi + EGFRi	2.0	2.0	SD	4.3	11.7	7.6	0.3
**Pt15**	Colorectal cancer	BRAFi + EGFRi + Iri	7.7*	11.3*	SD	9.6**	39.0	1.4	0.8*
**Pt16**	Colorectal cancer	BRAFi + EGFRi + Iri	6.3	10.6	PR	9.4	4.9	38.0	0.9
**Pt17**	Colorectal cancer	BRAFi + EGFRi + Iri	5.2	7.6	CR	1.1**	9.3	8.1	0.7

Abbreviations: i, inhibitor; Iri, irinotecan; BL, baseline; NE, not evaluable; ND, not detected; ctBRAFV600E, circulating BRAF V600E mutant tumor DNA; freq., frequency. * indicate patients still in treatment at the time of data cut-off and *[F] patients that were progression-free at that time. ** Indicate patients with several other cancer sites that were non-evaluable according to RECIST1.1 and thus not included in the overall tumor measure shown in this table.

Supplementary Table 3: Variants related to the MAPK pathway observed in tumor tissue and cfDNA. The gene variants were described using the *in silico* analysis software Ingenuity Knowledge Base (Qiagen) and the integrated software Alamut version 2.7 (http://www.interactive-biosoftware.com).

See Supplementary File 1

Supplementary Table 4: Extended list of cancer-associated variants identified by exome sequencing of tumor tissue DNA and cfDNA.Variant lists were exported fromIngenuity Variant Analysis version 5.1 and includes information on patient ID, sample type and variant type, coverage and frequency. Variants previously described in cancer are indicated by COSMIC ID and the number of times the variant has been described (including COSMIC and literature) recorded by the Ingenuity Knowledge database ("Variant Findings"). Furthermore, we included the Ingenuity variant classification. Abbreviations: Chr, Chromosome; Ref, Reference; Var, Variant.

See Supplementary File 2
