# Supplementary material for: Circulating tumor DNA as a marker of treatment response in BRAF V600E mutated non-melanoma solid tumors
Source: Oncotarget. 2018 Aug 24;9(66):32570–9. doi: 10.18632/oncotarget.25948 (PMC6135692; doi:10.18632/oncotarget.25948)
Supplement: Supplementary file 2 [file oncotarget-09-32570-s002.pdf]

Supplementary Table 3. Variants related to the MAPK pathway observed in tumor tissue and cfDNA. The gene variants were described using the *in silico* analysis software Ingenuity Knowledge Base (Qiagen) and the integrated software Alamut version 2.7 (http://www.interactive-biosoftware.com).

Patient ID	MAPK variant	Characterization of the variant
**Pt5 Bile duct Cancer**<br><br>**Tissue**	*BRAF* c.1799T>A, p.V600E (AF 0.23)	Oncogenic mutation in *BRAF* (B-Raf proto-oncogene, serine/threonine kinase) leading to constitutive activation of the protein kinase (COSMIC ID: 1131; 467, [1]).
	TP53 c.994-2A>C (AF 0.39)	Splice site disrupting mutation in *TP53* (tumor protein p53) leading to a shift in the reading frame and a premature stop codon. The mutation has previously been described as likely pathogenic in a range of databases and cancers (COSMIC ID: 3970338; 3970337).
	TGFBR2 c.383dupA, p.P129fs (AF 0.14)	Truncating mutation in the *TGFBR2* (transforming growth factor beta receptor 2) gene. The mutation has previously been described in cancer (COSMIC ID: 4613098; 4765129; 1172978; 300123) as a tumor suppressor gene involved in TGF-beta signalling regulating cell growth. Due to the location and the truncating nature of this mutation it might inactivate the pathway, leading to increased cell proliferation but functional studies are needed to clarify the role of this mutation.
**Pt5 Bile duct Cancer**<br><br>**Plasma**	*TAOK2* c.1155_1157delGGA, p.E392del (AF 0.11/0.04))	In-frame deletion in *TAOK2* encoding the TAO kinase 2. The mutation has previously been described in cancer (COSMIC ID: 25850690; 5850691), but the functional effect is unknown. The deletion is located outside the kinase domain and is observed in a high proportion of the general population (0.33%) making it unlikely to drive carcinogenesis.
	FGFR3 c.1636G>A, p.A546T (AF 0.035)	Missense variant in the gene encoding fibroblast growth factor receptor 3 (*FGFR3*). The variant is located in the kinase domain but has not previously been described in the literature or mutation databases (e.g. ClinVar and COSMIC). *In silico* predictions (Align-GVGD, SIFT) indicates a benign variant whereas MutationTaster indicates a disease-causing variant.
	MYC c.154_156del, p.Gln52del (AF 0.027)	In-frame deletion in the *MYC* proto-oncogene previously described in tumor tissue from patients with malignant melanoma treated with BRAFi therapy (COSMIC ID: 4704354). Activating alterations of MYC leads to altered transcription of target genes, but the function of this deletion is unknown.
	PTPRR c.1480C>A, p.L494I (AF 0.055)	Missense variant in the gene encoding the protein tyrosine phosphatase, receptor type R (*PTPRR*). The variant has previously been described in cancer (COSMIC ID: 6138165; 6138166) and silencing of the gene has been associated with colorectal cancer. *In silico* predictions (Align-GVGD, SIFT) indicates a benign variant whereas MutationTaster indicates a disease causing variant. Multiple transcript variants encoding different isoforms have been found for PTPRR and the functional effect of this variant is not known.
	CACNA1A c.6750_69del, p.Trp2251Glyfs (AF 0.021)	20-bp deletion in *CACNA1A* (calcium voltage-gated channel subunit alpha1A) leading to a shift in the reading frame. The deletion is located in the second last exon outside important

		functional domains. The variant is not previously described in the literature or cancer databases.
	BRAF c.1799T>A, p.V600E (AF 0.12/0.049)	Oncogenic mutation in *BRAF* (B-Raf proto-oncogene, serine/threonine kinase) leading to constitutive activation of the protein kinase (COSMIC ID: 1131; 467, [1]).
	TP53 c.994-2A>C (AF 0.20/0.056)	Splice site disrupting mutation in *TP53* (tumor protein p53) leading to a shift in the reading frame and a premature stop codon. The mutation has previously been described as likely pathogenic in a range of databases and cancers (COSMIC ID: 3970338; 3970337).
**Pt7 Colorectal Cancer**<br><br>**Plasma**	*CACNA1C* c.5611G>A, p.E1871K (AF 0.06)	Missense variant in a poorly described region of *CACNA1C* (calcium voltage-gated channel subunit alpha1C). *In silico* predictions (Align-GVGD, SIFT) indicates a benign variant. The variant is not previously described in the literature or mutation databases (e.g. ClinVar and COSMIC).
	MAP2K3 (MEK3) c.520_521insT, p.H203fs (AF 0.07)	Frameshift mutation in the protein kinase domain of the *MAPK3* (mitogen-activated protein kinase kinase 3) gene. Overexpression of MEK3 have been associated with malignancies, making a truncating mutation unlikely pathogenic. The variant is not previously described in literature or mutation databases (e.g. ClinVar and COSMIC).
	BRAF c.1799T>A, p.V600E (AF 0.10)	Oncogenic mutation in *BRAF* (B-Raf proto-oncogene, serine/threonine kinase) leading to constitutive activation of the protein kinase (COSMIC ID: 1131; 467, [1]).
**Pt9 Colorectal Cancer**<br><br>**Plasma**	*MAP2K1* (MEK1) c.173A>C, p.Q58P (AF 0.12)	Missense mutation in a conserved region of *MAP2K1* (mitogen-activated protein kinase kinase 1) with a previously described oncogenic variant at the neighboring position (p.K57T, rs1057519909) and variants (p.F53Y and p.V60E) observed in BRAFi-resistant melanoma cancers [33]. *In silico* predictions (Align-GVGD, SIFT) indicates a benign variant whereas MutationTaster indicates a disease causing variant. The variant is not previously described in the literature or mutation databases (e.g. ClinVar and COSMIC) and functional studies are lacking.
	BRAF c.1799T>A, p.V600E (AF 0.31)	Oncogenic mutation in *BRAF* (B-Raf proto-oncogene, serine/threonine kinase) leading to constitutive activation of the protein kinase (COSMIC ID: 1131; 467, [1]).
**Pt17 Colorectal Cancer**<br><br>**Plasma**	*RPS6KA4* c.1247C>T, p.A353V (AF 0.89)	Missense mutation in the protein kinase domain of the *RPS6KA4* (ribosomal protein S6 kinase A4) gene. The variant is not previously described in the literature or mutation databases (e.g. ClinVar and COSMIC) and i*n silico* predictions (Align-GVGD, SIFT) indicates a benign variant.
	BRAF c.1799T>A, p.V600E (AF 0.19)	Oncogenic mutation in *BRAF* (B-Raf proto-oncogene, serine/threonine kinase) leading to constitutive activation of the protein kinase (COSMIC ID: 1131; 467, [1]).
